# Role of Extracellular microRNAs in Sepsis-Induced Acute Lung Injury

**DOI:** 10.1155/2023/5509652

**Published:** 2023-06-19

**Authors:** Chenlu Xiong, Xuan Huang, Shibiao Chen, Yong Li

**Affiliations:** ^1^Department of Anesthesiology, Medical Center of Anesthesiology and Pain, The First Affiliated Hospital of Nanchang University, Nanchang, China; ^2^The National Engineering Research Center for Bioengineering Drugs and the Technologies, Institute of Translational Medicine, Nanchang University, Nanchang, China

## Abstract

Acute lung injury (ALI) is a life-threatening pathological disease characterized by the damage of pulmonary endothelial cells and epithelial cell barriers by uncontrolled inflammation. During sepsis-induced ALI, multiple cells cooperate and communicate with each other to respond to the stimulation of inflammatory factors. However, the underlying mechanisms of action have not been fully identified, and the modes of communication therein are also being investigated. Extracellular vesicles (EVs) are a heterogeneous population of spherical membrane structures released by almost all types of cells, containing various cellular components. EVs are primary transport vehicles for microRNAs (miRNAs), which play essential roles in physiological and pathological processes in ALI. EV miRNAs from different sources participated in regulating the biological function of pulmonary epithelial cells, endothelial cells, and phagocytes by transferring miRNA through EVs during ALI induced by sepsis, which has great potential diagnostic and therapeutic values. This study aims to summarize the role and mechanism of extracellular vesicle miRNAs from different cells in the regulation of sepsis-induced ALI. It provides ideas for further exploring the role of extracellular miRNA secreted by different cells in the ALI induced by sepsis, to make up for the deficiency of current understanding, and to explore the more optimal scheme for diagnosis and treatment of ALI.

## 1. Introduction

Acute lung injury (ALI) is a life-threatening pathological disease, which remains major cause of morbidity, mortality, and healthcare burden of critically ill patients [1]. ALI is characterized by pulmonary inflammation, damage to the alveolar–capillary barrier and hypoxemia [2]. The pathophysiology of sepsis-induced ALI has not been fully elucidated. There are many risk factors for ALI, such as severe shock, infection, mechanical injury, and so on, among which the most common risk factor is severe sepsis [3]. The lung is the initial and most vulnerable target organ during sepsis, and about 25%–50% of septic patients may develop ALI or even acute respiratory distress syndrome (ARDS) [4]. It is noteworthy that the mortality rate of sepsis-induced ALI is higher than that caused by other risk factors [5]. Despite major advances in supportive care recently, the mortality rate for patients with ALI has decreased over time but is still as high as 40% [6]. Undoubtedly, the identification of new therapeutic targets and preventive approaches that are innovative, safe, and effective is crucial for the successful treatment of sepsis-induced ALI.

Extracellular vesicles (EVs) have recently emerged as key mediators in the pathogenesis of sepsis and ALI [7, 8]. The potential of harnessing EVs in the diagnosis and treatment of diseases is now being actively explored [9]. EV is a heterogeneous group of endogenous nanosized spherical membrane structures released by almost all types of cells, which is initially considered as a process of discarding membrane proteins in cells [10]. With the progress of research, it has been found that EVs are closely related to intercellular material transmission and signal communication, which can be released into a variety of bodily fluids including blood, urine, saliva, and bronchoalveolar lavage fluid (BALF) [11]. After ALI, there are abundant EVs detected in BALF, which originated from different cells. Furthermore, BALF EVs differ significantly in lung injury caused by sterile or infectious stimuli [12]. In the lipopolysaccharide (LPS)-induced ALI model, EVs were packaged with microRNA (miRNA) and cytokines, and then secreted to BALF [13].

EVs are composed of small lipid bilayers surrounding vesicles, which contain cellular components such as cytosolic proteins, DNA, and RNA [14]. Among them, there is a large amount of RNA in EVs, which can exchange genetic information between cells via carrying out intercellular communication by transferring messenger RNA and miRNA [15]. Extracellular miRNA plays a crucial role in the occurrence, maintenance, and resolution of a variety of diseases including ALI, which can be used as a new diagnostic and therapeutic target for various noncancer diseases (such as metabolic abnormalities) [16, 17]. Consequently, a growing number of studies have focused on the roles of extracellular miRNAs in lung injury and inflammation.

Here, we intend to update the latest knowledge about the roles of extracellular miRNAs in sepsis-induced ALI (Table 1), and discuss their diagnostic and therapeutic potential as facilitators of cell communication via miRNA as well as the relevance of microorganism-derived EVs.

## 2. Extracellular Vesicles (EVs)

**Figure 1:**
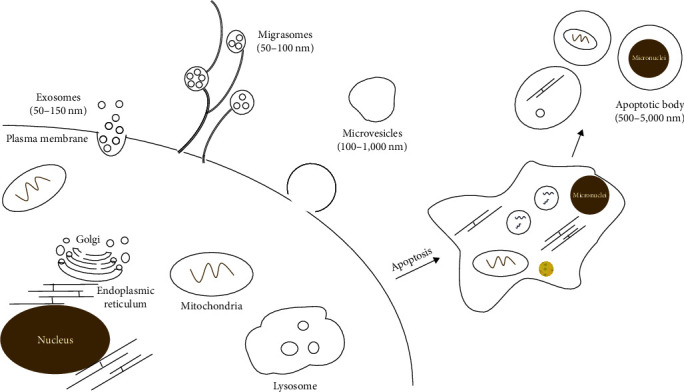
Subtypes of extracellular vesicles (EVs).

EVs are composed of small lipid bilayers around vesicles with diameters ranging from 40 to 1,000 nm [32]. Three main subtypes of EVs have been classified based on the mechanisms of formation, the membrane compositions, and the size of EVs, including exosomes (50–150 nm), macrovesicles (100–1,000 nm), and apoptotic bodies (500–5,000 nm) [33]. Most notably, migrasomes are a recently discovered type of EVs with diameters of about 50–100 nm, which are characteristically generated along retraction fibers in migrating cells [34] (Figure 1). Although several comparative proteomics studies have provided a list of proteins that may be specific for the identified EV subtypes, EV isolation methods to date only enable enrichment but not distinct separation of these EV subpopulations [35], thus the current article collectively refers to all vesicles released by cells as EVs. These EVs transfer cytosolic proteins, nucleic acids, or lipids to target cells [14], inducing transferring cellular components and changes in target-cell phenotypes and functions [36, p. 96]. According to the type of secretory cells, a group of cell type-specific proteins will be displayed in the EVs, which explain their specific fate and function.

The content of EVs is influenced by the environmental conditions and cell type, and other factors (e.g., infection or artificial expression of molecules), and hence it will directly affect the fate and function of EVs [37]. EV is involved in inflammation inhibition, immune regulation, transportation, and transmission of genetic information [38–40]. For instance, after ALI, there are abundant EVs detected in BALF, which originated from different cells, when subjected to the addition of plasma obtained from septic patients, or, the addition of pure LPS. In the LPS-induced ALI model, EVs were secreted in BALF, packaged with miRNA and cytokines, suggesting a complicated relationship between several cellular pathways occurring in sepsis [13].

Although, the components of EVs during the development of lung injury and inflammation are highly regulated, such as proteins, lipids, DNA, and RNA molecules, only RNA compositions are robustly increased in each EVs after normalization with the number of EVs. It seems that different miRNAs containing EVs play specific functional roles after specific stimuli [41]. The function and mechanism of these EVs-containing miRNAs in sepsis remain unclear, which may have the great potential to be diagnostic biomarkers and therapeutic targets [42].

## 3. Extracellular miRNAs

In 2007, Valadi et al. [43] found that both mRNA and miRNA existed in exosomes of mast cells, which can be delivered into another cell through a specific and regulated process and be functional in this new location. This genetic communication between cells may occur in the extracellular microenvironment but could also occur at a distance by traffic of exosomes through the systemic circulation in a similar way to hormones [44]. More importantly, if exosomes deliver a specific mRNA or miRNA, it may be more effective in affecting the recipient cell by modifying the protein production and gene expression of the recipient cell. Furthermore, those extracellular miRNAs bypass the transcriptional control of receptor cells through the intercellular transfer of foreign bodies and regulate the expression of target genes in receptor cells of different tissues [45]. As noncoding RNAs (ncRNAs), miRNAs are enriched in exosomes while others are barely present, which may share the same specific sequence (such as the EXO motif), suggesting a potential regulatory mechanism for the sorting of specific sets of miRNAs into exosomes [45].

miRNA is highly stable due to its small size compared with long mRNAs, which are identified to be differentially expressed in different stages of the disease and contribute to the diagnosis, treatment determination, and prognosis [46]. EVs containing miRNAs may be the emerging targets for developing novel therapeutic and diagnostic agents. As novel endocrine factors, extracellular miRNA can be used as a new diagnostic and therapeutic target for various noncancer diseases (such as metabolic disease), which plays a crucial role in the occurrence, maintenance, and resolution of a variety of diseases, including ALI [47, 48].

## 4. Extracellular miRNAs in Sepsis-Induced ALI

### 4.1. Extracellular miRNAs Derived from Mesenchymal Stem Cells

In severe bacterial pneumonia, microbubbles derived from human mesenchymal stem cells (MSCs) are as effective as parental stem cells [49]. EVs of human bone marrow-derived mesenchymal stem cells (hBMSCs) have been studied as therapeutic methods in various ALI models because they can reduce inflammation, lung permeability, and bacterial pneumonia [49–51]. MSC-EVs contain a large amount of RNA, including miRNAs [52, 53], which can not only regulate gene expression and transcription but also transfer into target cells and mediate gene expression and regulate cell function [43].

Accumulated evidence has revealed that MSC can play a role in sepsis-induced ALI [54, 55] because they can secrete paracrine factors such as growth factors, anti-inflammatory cytokines, and antimicrobial peptides [56–58]. Keratinocyte growth factor (KGF) is a paracrine factor secreted by hBMSCs. It has been proved that KGF can repair ALI induced by *Escherichia coli* endotoxin and bacteria perfused into the human lung in vitro, partially restore lung protein permeability, and reduce alveolar inflammation [55, 59]. MSC can transfer miR-30b-3p into mouse alveolar epithelial cells (AECs) through exosomes to inhibit the expression of SAA3 and increase the expression of KGF, thereby promoting the proliferation of LPS-treated AECs and inhibiting their apoptosis, which plays a role in reducing the inflammatory response and repairing endothelial cells against ALI [18]. Hao et al. [22] reported that bone marrow MSCs secreted EVs carrying miR-145 and transferred them to macrophages, which inhibited the activity of MRP1, thus enhancing the production and antibacterial activity of LTB4 through LTB4/BLT1 signal transduction, and increased the phagocytosis of macrophage cells to *E. coli*.

Autophagy is a powerful degradation pathway that plays a crucial role in various diseases [60]. Exosomes released by human umbilical cord mesenchymal stem cells (HucMSCs) induce autophagy in LPS-induced ALI, protecting against ALI [19]. The overexpression of miR-377-3p in HucMSCs exosomes can reduce LPS-induced ALI by targeting the inhibition of mTOR regulatory-related protein which stimulated the autophagy of LPS-treated human alveolar epithelial cells [19].

MSCs from various tissues and adipose-derived mesenchymal stem cells (ADSCs) are a group of attractive pluripotent MSCs due to their abundance and easy accessibility [61]. Compared with bone marrow-derived MSCs and ADSCs are more easily obtained by minimally invasive methods. In sepsis, dead cells release extracellular histones, which can induce endothelial injury and lead to ALI and multiple organ failure (MOF) [62–64]. MiR-126 was significantly increased in histone-treated ADSCs and exosomes derived from histone-treated ADSCs, which can activate PI3K/Akt signal and inhibit endothelial cell apoptosis [20]. Therefore, ADSCs can indirectly protect endothelial cells through the paracrine effect of exosomes.

### 4.2. Extracellular miRNAs Derived from Macrophage

Macrophages, the first responders of all immunoregulatory cells, are involved in the initiation and progression of lung inflammation and play a central role in the pathogenesis of ALI, which could be a new biomarker and treatment of ALI [65, 66]. Macrophage extracellular vesicle-mediated miRNA may provide a new therapeutic strategy in a cell-specific manner [67]. Compared with other delivery methods, microvesicles have some potential advantages as a carrier for delivering exogenous nucleotides [68]. Macrophages can be obtained from the blood of the host. Thus Zhang et al. [23] thought that the microbubbles secreted by macrophages to deliver miRNA molecules as therapeutic agents may trigger a less immune response, increase efficacy, and have fewer nontarget effects.

Infectious stimuli can increase miR-223/142 levels in microvesicles secreted by macrophages, and thus miR-223/142 in the circulation may serve as a potential marker to indicate lung macrophage activation or inflammation, and predict lung inflammation and its changes after bacterial infection [23]. Intracellular miR-223/142 was delivered via microvesicle-mediated delivery, and miR-223 and miR-142 synergistically inhibited activation of the NLRP3 inflammasome in macrophages by inhibiting NLRP3 and ASC, respectively, leading to suppression of lung inflammation [23]. MiR-155 belongs to a multifunctional miRNA family and has been reported to be associated with multifactorial-induced lung inflammation [69]. Macrophage-derived miR-155 mediates the expression of inflammatory factors in LPS-induced ALI through SOCS-1 and promotes inflammation [24].

Macrophage polarization occurs when macrophages phenotypically mount a specific phenotype and functional response to different pathophysiological conditions and surrounding microenvironments [70]. In the rehabilitation phase of ALI/ARDS, recruited macrophages then shift from the M1 to the M2 phenotype [71]. Wang et al. [21] found miR-27a-3p carried in EVs transferred from bone marrow MSCs to macrophages, which induced M2 macrophage polarization, inhibited the expression of NFKB1, and alleviated LPS-induced lung injury. Phagocytosis of dying cells and pathogens from a host by macrophages is also an efficient process for the resolution of inflammation [72, 73].

### 4.3. Extracellular miRNAs Derived from Polymorphonuclear Neutrophils

During ALI, inflammatory cells, mainly polymorphonuclear neutrophils, are in close contact with AECs. Many researchers have studied the intercellular communication of neutrophils in ALI, including paracrine cross talk between neutrophils and lung parenchymal cells [74]. Neutrophils secrete EVs carrying bioactive substances, including miRNA, which mediate intercellular communication and horizontal transfer of genetic material [75]. Neudecker et al. [25] found that miR-223 can transfer from neutrophils to lung epithelial cells through EVs, mediate PARP-1 inhibition, and has anti-inflammatory and protective effects on ALI. PARP-1, a miR-223 target gene in lung epithelial cells, is related to inflammation and ischemia-reperfusion tissue injury. miR-223 limits excessive lung inflammation during ALI by inhibiting PARP-1.

### 4.4. Extracellular miRNAs Derived from Endothelial Progenitor Cells and Endothelial Cells

Endothelial dysfunction is the pathophysiologic basis of ALI syndromes with dysfunction in several aspects, including coagulation, fibrinolysis, permeability, leukocyte recruitment, and vascular tone [76]. The mechanisms supporting these functions are highly complex, but some independent regulatory factors can be regulated explicitly for some independent factors without damaging other protective innate immune responses [77].

Endothelial progenitor cells (EPCs) can promote the proliferation, migration, and tube formation of endothelial cells, thereby reducing vascular leakage and inflammation, and improving bacterial clearance in sepsis-induced lung injury, pneumonia, and ALI [78, 79]. EPCs can migrate from bone marrow and then locate at the site of tissue injury, which has been studied as a possible therapeutic approach [80]. Moreover, it can secrete exosomes for intercellular communication to attenuate LPS-induced lung injury [28].

Wu et al. [26] suggested that EPCs secrete exosomes to transfer miR-126 to endothelial cells, and miR-126 regulates endothelial cell proliferation, migration, and tube formation by targeting spred-1 to activate Raf/ERK signaling. Jin et al. [27] believed that EPCs increase the expression of miR-10a/b-5p in lung tissue and pulmonary microvascular endothelial cells of ALI induced by LPS. MiR-10a/b-5p reduces the protein level of adam15 and promotes the proliferation of multisegmented microvascular endothelial cells induced by LPS. In addition, EPCs play a therapeutic role in ALI by promoting LPS-induced MPMVEC proliferation by regulating the miR-10a/b-5p/adam15 axis. Zhou et al. [28] suggested that both miR-126-3p and 5p in endothelial progenitor cell exosomes could increase the expression of tight junction proteins, including claudin1, claudin4, and occludin, by inhibiting phosphoinositide-3-kinase regulatory subunit 2 (pik3r2) and HMGB1 to restore alveolar barrier integrity and attenuate alveolar edema and lung injury. The overexpression of miR-126-3p could target pik3r2, whereas the overexpression of miR-126-5p inhibited inflammatory factor HMGB1 and permeability factor VEGF*α*. In addition, miR-126-5p delivered by exosomes inhibits VEGF*α* expression, further reducing ALI-induced lung permeability decline.

Endothelial cells can also secrete exosomes to attenuate sepsis-induced ALI. Jiang et al. [29] demonstrated that miR-125b-5p was upregulated in endothelial cell-derived exosomes to protect sepsis-induced ALI by inhibiting TOP2A and inflammatory responses in lung tissues of ALI mice. Amplified miR-125b-5p promoted the expression of vascular endothelial growth factor in lung tissue while decreasing vascular endothelial growth factor levels in ALI mice serum. Exosomes and exosomal miR-125b-5p (Exo-miR-125b-5p) also inhibited apoptosis in mice with ALI lung tissue.

### 4.5. Extracellular miRNAs Derived from Lung Epithelial Cell

Common features of ALI/ARDS include a solid inflammatory response in the lung parenchyma, severe damage of epithelial and endothelial cell barriers leading to alveolar edema, decreased lung compliance, impaired gas exchange, and hypoxemia [81]. As the first line of defense against injury, the alveolar epithelium plays an essential role in maintaining lung integrity and function during the development of ALI [82]. Liu et al. [30] showed that AECs exposed to LPS or sepsis released more exosomes than normal AECs, and there was a significant difference in the expression profile of miRNA in exosomes compared with the control group, in which miR-92a-3p was significantly increased in the exosomes of AECs after LPS treatment. The exosomes produced by LPS-treated AECs can promote the activation of alveolar macrophages and enhance the inflammatory response of alveolar macrophages. Macrophages activated by miR-92a-3p in the exosomes released by AECs have specific effects on lung injury, but the exact mechanism of miR-92a-3p-induced macrophage activation is unclear. Pulmonary epithelial-derived vesicles regulate macrophage migration and microvascular function by delivering miRNA-17/221-induced integrin *β*1 recycling. The transmission of miRNA in EVs from lung epithelial cells may provide a new way for the treatment of ALI [31].

## 5. Conclusion

**Figure 2:**
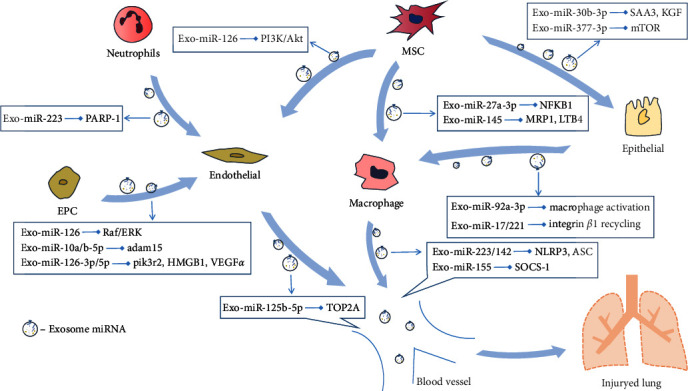
Extracellular microRNAs in sepsis-induced ALI. During sepsis-induced ALI, Exo-miRs released from different cells (such as neutrophils, MSCs, and EPCs) were transported by extracellular vesicles (EVs) and transferred to recipient cells (such as macrophage, endothelial cells, and epithelial cells), where they regulate posttranscriptional gene expression.

This review aimed to summarize the mechanisms by which different cells regulate the repair of ALI through extracellular vesicle transfer of miRNAs and to provide ideas for further exploring the role of extracellular miRNAs secreted by different cells in sepsis-induced ALI (Figure 2). This review also aimed to make up for the lack of existing knowledge and explore a better scheme for the diagnosis and treatment of ALI.

## Figures and Tables

**Table 1:** Exo-miRNA involved in sepsis-induced ALI.

Derived from cell types	Exo-miRNA	Target cell types	Mechanisms	Reference no.
Mesenchymal stem cells	Exo-mir-30b-3p	Epithelial cells	Cell membrane repair	[18]
	Exo-mir-377-3p	Epithelial cells	RPTOR/autophagy	[19]
	Exo-miR-126	Endothelial cells	PI3K/Akt signaling/apoptosis	[20]
	Exo-miR-27a-3p	Macrophage	Macrophage polarization/NF-*k*B signalling	[21]
	Exo-miR-145	Macrophage	Macrophage phagocytosis	[22]

Macrophage	Exo-miR-223/142	Macrophage	NLRP3 inflammasome activity	[23]
	Exo-miR-155		SOCS-1 signaling	[24]

Neutrophils	Exo-miR-223	Epithelial cell	PARP-1 inhibition	[25]

Endothelial progenitor cells	Exo-miR-126	Endothelial cells	Raf/ERK signaling	[26]
	Exo-miR-10a/b-5p	Endothelial cells	miR-10a/b-5p/adam15 axis	[27]
	Exo-miR-126-3p/5p	Endothelial cells	Restore lung permeability	[28]

Endothelial cells	Exo-miR-125b-5p		miR-125b-5p/TOP2A/VEGF axis	[29]

Epithelial cells	Exo-miR-92a-3p	Alveolar macrophages	NF-*k*B signalling	[30]
	Exo-miR-17/221	Macrophage	Integrin *β*1 recycling	[31]

## Data Availability

Data sharing is not applicable to this article as no new data were created or analyzed in this study.
